# Sebaceomas in a Muir–Torre-like Phenotype in a Patient with MUTYH-Associated Polyposis

**DOI:** 10.3390/dermatopathology11010011

**Published:** 2024-03-04

**Authors:** Julia Guarrera, James C. Prezzano, Kathleen A. Mannava

**Affiliations:** 1 Norton College of Medicine, SUNY Upstate Medical University, Syracuse, NY 13210, USA; 2 Fayetteville Dermatology, Fayetteville, NY 13066, USA; 3 Department of Pathology & Laboratory Medicine, Department of Dermatology, University of Rochester School of Medicine and Dentistry, Rochester, NY 14620, USA

**Keywords:** Lynch syndrome, MUTYH associated polyposis, Muire–Torre syndrome, sebaceoma, colorectal cancer, sebaceous neoplasm

## Abstract

This case report describes a case of a patient with MUTYH-associated polyposis (MAP), who presented with multiple sebaceomas in a Muir–Torre-like phenotype. MAP is caused by mutations in MUTYH, a base excision repair gene responsible for detecting and repairing the 8-oxo-G:A transversion caused by reactive oxygen species. MAP is associated with an increased risk of developing adenomatous polyps and colorectal cancer. Muir–Torre syndrome is a clinical phenotype of Lynch syndrome, which presents with multiple cutaneous sebaceous neoplasms. Lynch syndrome, like MAP, increases the likelihood of developing colorectal cancer but with a different pathogenesis and mode of inheritance. This case demonstrates that in a patient presenting with multiple sebaceous neoplasms, further workup and genetic testing may be indicated, not only for Muir–Torre and Lynch syndrome but also for MAP.

## 1. Introduction

MUTYH Associated Polyposis (MAP) is caused by mutations in the MUTYH gene, which is a base excision repair gene responsible for detecting and repairing the 8-oxo-G:A transversion caused by reactive oxygen species [1]. MAP is inherited in an autosomal recessive pattern and is associated with an increased risk of developing many adenomatous polyps, and subsequently, colorectal cancer [1,2]. Lynch syndrome is another inherited disorder, also referred to as hereditary non-polyposis colorectal cancer. Patients with Lynch syndrome have a higher likelihood of developing colorectal cancer as well as other malignancies, like endometrial and ovarian cancers [3]. Unlike MAP, Lynch syndrome has an autosomal dominant pattern of inheritance and is caused by a mutation in a mismatch repair gene. Lynch syndrome is also associated with high microsatellite instability [3]. Muir–Torre syndrome is a clinical phenotype of Lynch syndrome, which presents with multiple sebaceous neoplasms on the skin. In this case, we present a patient with MAP, who presented with multiple sebaceomas in a Muir–Torre-like phenotype.

## 2. Case Report

**Figure 1:**
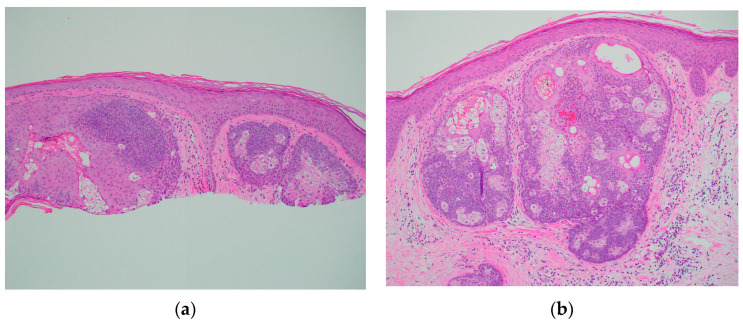
Skin shave biopsy showing sebaceomas on H&E. (**a**) ×10 Forehead; (**b**) ×10 Chest.

**Figure 2:**
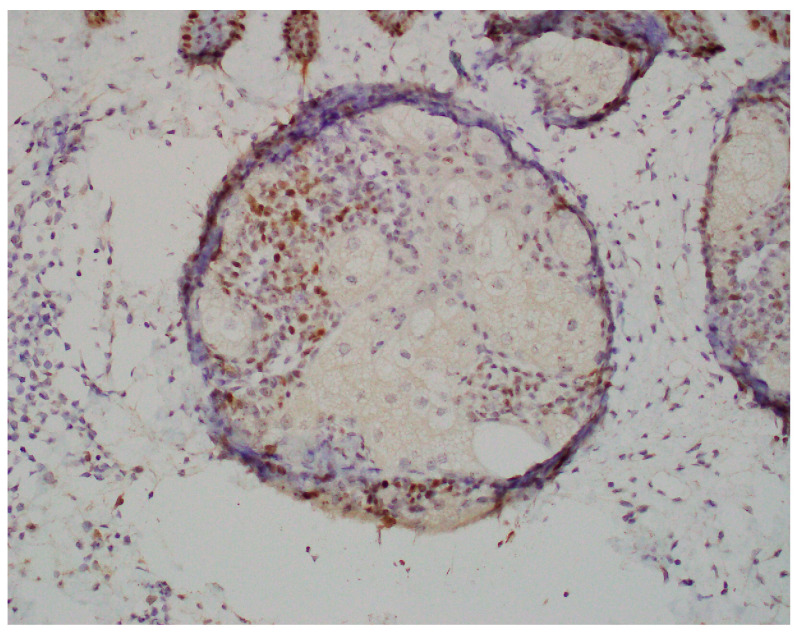
Sebaceoma from the chest showing partial loss of MSH6.

A 67-year-old Caucasian man presented to the dermatology clinic for a routine full-body skin exam. Upon examination, the patient had two flesh-colored smooth appearing papules, one on the right central forehead and one on the right medial inferior chest. Both lesions appeared to be clinically suspicious for basal cell carcinoma vs. sebaceous hyperplasia. In total, two shave biopsies were performed. In both specimens, histologic examination showed well-demarcated intradermal sebaceous neoplasms, composed of more than 50 percent germinative sebocytes, consistent with sebaceomas (Figure 1). Immunohistochemical staining for DNA mismatch repair proteins was performed. Both sebaceomas showed a partial loss of expression of MSH6 (Figure 2). The expression of MLH1, MSH2, and PMS2 was retained. It was noted in the pathology report that multiple sebaceous neoplasms and a loss of expression of DNA mismatch repair proteins may be seen in the setting of Muir–Torre syndrome, and further clinical correlation was advised. When the report was discussed with the patient, he reported that he had a history of a total colectomy. The patient had a long history of many tubular adenomas, which eventually led to a total abdominal colectomy. The patient had participated in further genetic testing, which revealed an autosomal recessive colorectal polyposis associated with a mutation in the MUTYH gene. The genetic testing also specifically identified a p.G396D gene mutation, which is one of the two most common mutations associated with MAP.

## 3. Discussion

MAP is an autosomal recessive inherited disease that increases the risk of developing adenomatous polyps and colorectal cancer [1]. MAP is caused by biallelic germline mutations in the MUTYH gene, which is a base excision repair enzyme used to repair the 8-oxo-G:A transversion caused by reactive oxygen species [1]. This transversion can lead to the development of colorectal polyps. The two common mutations in the MUTYH gene associated with MAP are the Y179C and G396D mutations [4]. Biallelic carriers are at a high risk of developing colorectal cancer, and genetic testing may be recommended for the patient and family members. Colorectal cancer in patients with MAP is often right-sided, and right-sided colorectal cancer has been shown to present later on in disease and have a worse prognosis and a lower survival rate than left-sided colon cancer [5,6]. Patients with MAP receive frequent colonoscopies to detect polyps before they progress, lowering the risk of colorectal cancer [4]. Patients may need more radical surgeries like abdominal colectomies, as our patient had, depending on when MAP is discovered and if multiple polyps or cancer is present. Earlier detection of this condition is important for the patient’s health. While Lynch syndrome is known to be associated with Muir–Torre, MAP has also been associated with sebaceous neoplasms and other cases have been reported in which patients with MAP present with sebaceous neoplasms in a Muir–Torre-like phenotype [7]. Muir–Torre syndrome is a clinical phenotype of Lynch syndrome, which is also known as hereditary non-polyposis colorectal cancer. Lynch syndrome also increases the likelihood of developing colorectal cancer as well, as other cancers including endometrial and ovarian, but differs in presentation from MAP [4]. Lynch syndrome is inherited in an autosomal-dominant fashion and differs in the type of genes involved [3]. Lynch syndrome is associated with mutations in DNA mismatch repair proteins [3]. The most common repair protein mutations affected are MLH1, MSH2, MSH6, and PMS2 [4].

Muir–Torre with Lynch syndrome often presents clinically with numerous cutaneous sebaceous neoplasms. These include sebaceous adenomas, sebaceous carcinomas, and sebaceomas [8]. Keratoacanthomas with sebaceous differentiation, as well as basal cell carcinomas with sebaceous differentiation, are also related [4]. Sebaceous adenomas are often the most common tumor [9]. The sebaceous lesions are most commonly found on the face, neck, and trunk regions, and may present as solitary or in multiples [8]. These lesions are often asymptomatic, flesh to yellow-colored papules on the skin, and are more commonly seen in men [8,9]. Histologically, lesions often show the same variants in the MSH2, MSH6, MLH1, and PMS2 genes as in Lynch syndrome [8]. Both of our patient’s sebaceomas were consistent with a partial loss of MSH6. These genes are mismatch repair genes that correct DNA replication errors [10]. Once Muir–Torre is discovered, patients are recommended to be screened annually due to the high association with Lynch syndrome and its relation to internal malignancy [4]. Additionally, patients should also be screened for cutaneous neoplasms as well. Benign sebaceous lesions do not require any treatment, but any sebaceous neoplasms with cancerous potential should be excised. The time between the initial presentation of the sebaceous lesion and internal malignancy varies. Some patients may develop a malignancy before or after a Muir–Torre diagnosis, which is why it is important to diagnose the condition early and set the patient up with further testing and monitoring [11].

Although Muir–Torre syndrome associated with MAP is less common than its association with Lynch syndrome, previous case reports have presented cases of sebaceous adenomas, sebaceous epitheliomas, and sebaceous carcinomas with a Muir–Torre-like phenotype in patients with MAP [7]. Our patient presented differently with sebaceomas. MAP cases with a Muir–Torre presentation have been classified as Muir–Torre syndrome II [4,9]. Muir–Torre syndrome II has been noted to make up 35% of tumors associated with Muir–Torre syndrome [4,9]. Like in MAP, it has autosomal recessive inheritance, and it is not associated with microsatellite instability seen in Muir–Torre syndrome I, which is associated with Lynch Syndrome [4,9]. In our case, our patient had MAP and presented with sebaceomas that were consistent with Muir–Torre syndrome. The sebaceomas showed a partial loss of MSH6, which is a less common variant [10]. Our patient had a confirmed history and testing for MAP and had already undergone a colectomy due to numerous polyps. In practice, however, if patients present with multiple sebaceous neoplasms, they should be tested for Muir–Torre syndrome by immunohistochemical staining and further genetic workup. Not only should testing be completed for Lynch Syndrome, but it is important for clinicians to consider ordering testing for MAP as it has been shown in case reports that MAP and Lynch syndrome may both appear phenotypically similar on the skin through Muir–Torre syndrome. MAP commonly presents in adulthood, and patients may be asymptomatic and have not had a prior colonoscopy. Further genetic testing for biopsied lesions that are suspicious for Muir–Torre may aid in earlier treatments, monitoring, and a better outcome for patients. If MAP is detected sooner, it may provide earlier detection of pre-cancerous colorectal polyps and colorectal cancer in patients whose skin presentation was their first sign of disease.

## 4. Conclusions

Patients with MAP presenting with Muir–Torre-like syndrome have been reported and this case further demonstrates the importance of biopsying suspicious lesions and completing further genetic testing. While this patient was already diagnosed with MAP, a Muir–Torre-like presentation may be the first sign of disease in some patients. Patients should also not only undergo testing only for Lynch syndrome but also for MAP to ensure that an accurate diagnosis is made. Distinguishing between MAP and Lynch syndrome is important in regard to increasing patient awareness of their specific genetic condition and their further cancer screening efforts. It also allows patients to inform and discuss with family members who may also be affected, as the mode of inheritance differs between syndromes.

## Data Availability

Data are contained within the article. The original contributions presented in the study are included in the article, further inquiries can be directed to the corresponding author.
